# The Pharmacological Profile of a Novel Highly Potent Bisphosphonate, OX14 (1‐Fluoro‐2‐(Imidazo‐[1,2‐α]Pyridin‐3‐yl)‐Ethyl‐Bisphosphonate)

**DOI:** 10.1002/jbmr.3138

**Published:** 2017-04-21

**Authors:** Michelle A Lawson, Frank H Ebetino, Adam Mazur, Andrew D Chantry, Julia Paton‐Hough, Holly R Evans, Darren Lath, Maria K Tsoumpra, Mark W Lundy, Roy LM Dobson, Michael Quijano, Aaron A Kwaasi, James E Dunford, Xuchen Duan, James T Triffitt, Gwyn Jeans, R Graham G Russell

**Affiliations:** ^1^ Department of Oncology and Metabolism Medical School University of Sheffield UK; ^2^ Mellanby Centre for Bone Research Medical School University of Sheffield UK; ^3^ Department of Chemistry University of Rochester Rochester NY USA; ^4^ TWI Chem LLC Mason OH USA; ^5^ Department of Anatomy and Cell Biology Indiana University Indianapolis IN USA; ^6^ Procter & Gamble Mason OH USA; ^7^ Nuffield Department of Orthopaedics, Rheumatology and Musculoskeletal Sciences The Oxford University Institute of Musculoskeletal Sciences The Botnar Research Centre Nuffield Orthopaedic Centre Oxford UK

**Keywords:** ANTIRESORPTIVES, TUMOR‐INDUCED BONE DISEASE, BONE HISTOMORPHOMETRY, BONE μCT, MULTIPLE MYELOMA

## Abstract

Bisphosphonates are widely used in the treatment of clinical disorders characterized by increased bone resorption, including osteoporosis, Paget's disease, and the skeletal complications of malignancy. The antiresorptive potency of the nitrogen‐containing bisphosphonates on bone in vivo is now recognized to depend upon two key properties, namely mineral binding affinity and inhibitory activity on farnesyl pyrophosphate synthase (FPPS), and these properties vary independently of each other in individual bisphosphonates. The better understanding of structure activity relationships among the bisphosphonates has enabled us to design a series of novel bisphosphonates with a range of mineral binding properties and antiresorptive potencies. Among these is a highly potent bisphosphonate, 1‐fluoro‐2‐(imidazo‐[1,2 alpha]pyridin‐3‐yl)‐ethyl‐bisphosphonate, also known as OX14, which is a strong inhibitor of FPPS, but has lower binding affinity for bone mineral than most of the commonly studied bisphosphonates. The aim of this work was to characterize OX14 pharmacologically in relation to several of the bisphosphonates currently used clinically. When OX14 was compared to zoledronate (ZOL), risedronate (RIS), and minodronate (MIN), it was as potent at inhibiting FPPS in vitro but had significantly lower binding affinity to hydroxyapatite (HAP) columns than ALN, ZOL, RIS, and MIN. When injected i.v. into growing Sprague Dawley rats, OX14 was excreted into the urine to a greater extent than the other bisphosphonates, indicating reduced short‐term skeletal uptake and retention. In studies in both Sprague Dawley rats and C57BL/6J mice, OX14 inhibited bone resorption, with an antiresorptive potency equivalent to or greater than the comparator bisphosphonates. In the JJN3‐NSG murine model of myeloma‐induced bone disease, OX14 significantly prevented the formation of osteolytic lesions (*p* < 0.05). In summary, OX14 is a new, highly potent bisphosphonate with lower bone binding affinity than other clinically relevant bisphosphonates. This renders OX14 an interesting potential candidate for further development for its potential skeletal and nonskeletal benefits. © 2017 The Authors. *Journal of Bone and Mineral Research* Published by Wiley Periodicals Inc.

## Introduction

Bisphosphonates continue to be the major drugs used in the prevention and treatment of diseases or conditions characterized by increased bone resorption, including osteoporosis and Paget's disease. They are also used to prevent the skeletal complications of malignancy in breast, prostate, and other cancers, as well as in multiple myeloma, and may increase the survival of patients with some of these cancers.^1^, ^2^ Zoledronate (ZOL, also often known as zoledronic acid, ZA) is given intravenously (i.v.) rather than orally, provides the standard of care for many of these conditions, and is used especially in bone oncology.

The first bisphosphonates to be developed for clinical use, etidronate, clodronate, pamidronate, and alendronate (ALN), were already known chemical compounds.^3^ Subsequently, medicinal chemists synthesized literally hundreds of compounds from which the next generation of successful bisphosphonates were chosen, notably risedronate (RIS), ibandronate (IBN), and ZOL.^4^, ^5^ These bisphosphonates emerged from screening programs and were selected based on their potency and safety profiles, but in an era before the detailed molecular mechanisms of action of bisphosphonates were elucidated.

The antiresorptive potency of the nitrogen‐containing bisphosphonates on bone in vivo is now recognized to depend upon two key properties, namely mineral binding affinity and inhibitory activity on farnesyl pyrophosphate synthase (FPPS); these properties vary independently of each other in individual bisphosphonates.^6^

This better understanding of structure activity relationships among the bisphosphonates has enabled us to design a series of novel bisphosphonates with a range of mineral binding properties and inhibitory potencies on FPPS. During the past decade we have synthesized dozens of new bisphosphonates, and screened them for these properties. Among these is a highly potent bisphosphonate with reduced affinity for bone mineral, namely 1‐fluoro‐2‐(imidazo‐[1,2 alpha]pyridin‐3‐yl)‐ethyl‐bisphosphonate, also known as OX14^7^ (see the Supporting Materials and Methods for further details of synthesis and structure). The aim of the work described herein was to characterize OX14 pharmacologically, and to compare it to other bisphosphonates currently used clinically.

There are several reasons why bisphosphonates with reduced bone binding characteristics, but retaining strong inhibitory activity on FPPS, might have potential value. Such compounds, like RIS, have a more rapidly reversible effect on reducing bone turnover compared with longer‐acting bisphosphonates such as ALN and ZOL. This may have advantages under certain circumstances; eg, in treating women of childbearing years. Furthermore, the risk of some of the side effects that are often attributed to long‐term bisphosphonate treatment, such as osteonecrosis of the jaw^8^, ^9^, ^10^ and atypical femoral factures,^11^, ^12^, ^13^, ^14^ may be minimized. Within bone itself, a bisphosphonate that detaches from bone surfaces more readily may have greater effects on cells within the marrow microenvironment, including tumor cells, and may even have beneficial effects in more distal tissues. Indeed, bisphosphonate treatment has been associated with a range of apparently nonskeletal effects, including reductions in mortality,^15^, ^16^, ^17^ a reduction of myocardial infarctions,^18^ and improved survival of certain patient groups.^19^, ^20^, ^21^ These considerations provided us with the rationale for studying the pharmacological profile of OX14.

## Materials and Methods

### Ethics statement

All procedures involving mice were conducted at the University of Sheffield, UK, and were approved by the Home Office (PPL 40/3462) and the University of Sheffield's Animal Ethics Committee in accordance with the Animal [Scientific Procedures] Act 1986. All procedures involving rats were conducted at the Procter & Gamble Health Sciences Center, Mason, OH, USA. They were performed in accordance with the guidelines of Procter & Gamble Pharmaceuticals’ Institutional Animal Care and Use Committee, and meet the guidelines established by the Animal Welfare Act.

### Materials

The bisphosphonates were obtained from Procter and Gamble Pharmaceuticals (FHE, Mason, OH, USA), except for ALN, which was from Sigma (St. Louis, MO, USA), and OX14 (1‐fluoro‐2‐(imidazo‐[1,2‐α]pyridin‐3‐yl)‐ethyl‐bisphosphonate), which was synthesized by AM (see Supporting Materials and Methods for details^7^).

### Cell lines

The JJN3 myeloma cell line (originally derived from the bone marrow of a 57‐year‐old woman with plasma cell leukemia at diagnosis) was purchased from DSMZ (Braunschweig, Germany) and grown in RPMI medium, containing 10% FCS, 1% penicillin/streptomycin (100 U/100 µg/mL), 1% non‐essential amino acids (1×), and 1% sodium pyruvate (1 mM) at 37°C in 5% CO_2_ (Sigma–Aldrich, Dorset, UK). The cell line was genetically profiled by American Type Culture Collection (ATCC, Manassas, VA, USA) using short tandem repeat analysis to confirm its identity.

### Animals

Sprague Dawley rats, C57BL/6J and NOD/SCID‐γ (NSG, NOD.Cg‐Prkdcscid Il2rgtm1Wjl/SzJ) mice were purchased from Charles River Laboratories (Wilmington, MA, USA/Margate, UK). All animals were housed in cages under standard conditions (12‐hour light/dark cycle). NSG mice were housed in specific‐pathogen‐free conditions. All animals were healthy and pathogen‐free at the start of each study and once each study had commenced they were monitored daily for any unexpected adverse effects. Animals were housed in groups and the numbers per group were determined using power calculations based on previous studies in which reproducible statistical differences had been shown.^22^ The following power calculation shows *n* = 8 mice/group using the JJN3‐NSG model to see a 25% difference in tumor burden (by histological analysis) and a 40% difference in myeloma‐induced bone disease (by µCT analysis).

$$n=2{\left(SD\right)}^{2}\times f(\alpha ,\beta )/{\left(\Delta \right)}^{2}$$

$$n=2{\left(2.45\right)}^{2}\times 10.5/{\left(4\right)}^{2}=7.88\;\left(\text{mice per experimental group}\right)$$

where the significance level is 0.05%, the power level is 90%, the least practical difference between the groups is 25%, and estimated coefficient of variance SD/mean = 2.45/16 = 0.15. Similar calculations were used to determine group numbers in non–tumor‐bearing animals based on previously observed changes in bone (BMD or trabecular bone fraction [BV/TV]).

### Mineral binding affinity

The binding affinities of OX14, ALN, ZOL, RIS, and minodronate (MIN) were compared by measuring their differential rates of elution from hydroxyapatite (HAP) columns using a fast protein liquid chromatography (FPLC) system similar to that previously described.^23^

### FPPS inhibition assay

The inhibitory activity of OX14 was compared to ALN, ZOL, RIS, and MIN in a FPPS inhibition assay as previously described.^24^, ^25^ The IC_50_ values are based on preincubation protocols.

### In vivo evaluation of OX14 and comparator bisphosphonates as inhibitors of bone resorption based on metaphyseal BMD

The antiresorptive potency of OX14 was measured by its effect on increasing BMD compared to ALN, ZOL, RIS, and MIN in a growing rat model adapted from the Schenk assay.^26^, ^27^ These studies were performed at the Procter & Gamble Health Sciences Center, Mason, OH, USA from 2007 to 2010, as part of the program of screening new bisphosphonates. Male Sprague Dawley rats (6 weeks old, weighing 120 to 150 g) were randomized into groups (*n* = 6) based on body weight. All groups received treatment by subcutaneous (s.c.) injection at the same time daily for 7 days. The first treatment was on day 0, and the last treatment on day 6, with animals euthanized by cervical dislocation on day 7. BMD of the proximal metaphysis of the right tibia was analyzed using dual‐energy X‐ray analysis (DXA) using a Hologic QDR‐4500 densitometer (Hologic, Inc., Waltham, MA, USA). Efficacy of each bisphosphonate was expressed as percentage change from the vehicle control group. The dose that increased BMD 20% greater than the control was determined via a logistic dose‐response relationship (SAS Institute, Inc., Cary, NC, USA), which estimates an efficacy value using animals in the study, and for comparison of bisphosphonates across studies. Data from compounds run in multiple studies was averaged.

### In vivo evaluation of short‐term (24‐hour) skeletal retention of OX14 and comparator bisphosphonates, based on urinary excretion

It is known that bisphosphonates are rapidly taken up by the skeleton after parenteral administration, and the portion that is not bound to bone is cleared by the kidneys and appears in the urine within a few hours. This provides a convenient way of assessing skeletal uptake as the portion of an administered dose that is not excreted within 24 hours. Male Sprague Dawley rats (6 weeks old, weighing 120 to 150 g) were randomized into groups based on weight, and fitted with i.v. femoral catheters. Prior to i.v. dosing, each rat was given a single oral dose of 2.5 mL saline to increase initial urine output. Doses of ALN, ZOL, MIN, RIS, and OX14 (18 to 72 µg P/kg) were given in various combinations and relative concentrations via an i.v. femoral catheter, which was then flushed with 2.5 mL non‐heparinized saline. Rats were then immediately placed in metabolism caging and urine was collected for 0 to 24 hours. At the end of urine collection, the metabolism caging was rinsed with 10 mL of sterile water to collect any urine that had not yet reached the collection container. Collected urine was analyzed for the simultaneously administered bisphosphonates, by employing ion‐pairing, reverse‐phase, gradient HPLC, with tandem mass spectrometry detection. Briefly, urine samples were prepared by dilution in water containing 100 mM dimethylhexylamine (ion pairing reagent) and ^2^H_4_‐RIS was added as an internal standard. Prepared samples were injected onto a 2‐mm × 50‐mm C18 column and separated using a rapid water:acetonitrile gradient and constant modifier composition of 10 mM ammonium formate, 10 mM dimethylhexylamine, and 0.4% formic acid. Chromatographic effluent was passed into the ion source of a Sciex API5000 triple quadrupole mass spectrometer, operated in negative ion mode and employing selected‐reaction‐monitoring, for selective detection of each bisphosphonate. In this, the first quadrupole mass analyzer sequentially transmitted the [M‐H]^–^ ion corresponding to each targeted bisphosphonate into a collision cell, from which a bisphosphonate‐characteristic fragment ion (neutral loss of m/z 82) was selected to pass through the second mass analyzer, for detection. This approach enabled specific detection of each bisphosphonate, even in the presence of closely related compounds and high concentrations of urine matrix components, allowing simultaneous quantification of these co‐administered compounds. With appropriate sample dilution, accurate measurements were achieved over a 0.1‐μg/mL to 10‐μg/mL range, employing analytical calibration curves created from a cocktail containing each bisphosphonate, and the ^2^H_4_‐RIS internal standard. Measurement accuracy was assured by also analyzing quality control samples, consisting of naive rat urine, fortified with known concentrations of bisphosphonate reference standards. Analytical recoveries were consistently in the 95% to 105% range. Bisphosphonate concentrations measured in urine were multiplied by the mass of urine collected for each time‐segment, and these results were added together across the 24‐hour period for each rat, yielding the absolute urinary excretion for each bisphosphonate. The percentage excretion data for each bisphosphonate whether given individually or in combination was averaged from all studies performed (OX14, *n* = 6; ALN, *n* = 9; ZOL, *n* = 6; RIS, *n* = 42; and MIN, *n* = 3).

### In vivo OX14 dose response study in mice

Female C57BL6 mice (7 to 8 weeks old, weighing 18 to 24 g) were randomized into six groups (*n* = 8/group) based on weight and treated at the same time on each day twice a week s.c. with PBS (100 µL), ZOL (28.4 µg P/kg), RIS (27.1 µg P/kg), or three different doses of OX14 (23, 2.3, and 0.23 µg P/kg) for 3 weeks. All animals were then anesthetized (100% wt/vol isoflurane and 2% oxygen by inhalation) for cardiac bleeding and euthanized by cervical dislocation. Tibias were then assessed by µCT and histological analyzes as described below (see Assessment of bone integrity).

### An in vivo therapeutic study comparing OX14 to ZOL in a murine model of myeloma

Female NSG mice (7 to 8 weeks old, weighing 19 to 23 g) were randomized based on weight into four groups (*n* = 8/group). Group 1 was a non–tumor‐bearing control group (naive). Groups 2 to 4 were injected via the tail vein with 1 × 10^6^ JJN3 cells and then treated twice a week s.c. from the time of tumor cell injection for the duration of the study with vehicle control (PBS, group 2), ZOL (28.4 µg P/kg, group 3), or OX14 (23 µg P/kg, group 4). At the first signs of morbidity (after 3 weeks) all animals were anesthetized (100% wt/vol isoflurane and 2% oxygen by inhalation) for cardiac bleeding and euthanized by cervical dislocation. Bone disease and tumor burden were assessed as described below (see Analysis of tumour burden).

### Assessment of bone integrity

At euthanasia, the right tibias were dissected free of soft tissue and fixed in 10% formalin before µCT analysis was used to measure the percentage of BV/TV. Tibias were scanned on a Skyscan 1272 (Bruker, Kontich, Belgium) at 4.3 μm with a 0.7‐degree rotation step, reconstructed using NRecon (v. 1.6.9.4; Bruker) and analyzed using CTAn (v. 1.12; Bruker). The number and area of cortical bone lesions (pixels^2^) were assessed by taking the µCT datasets, removing the trabecular bone and then volume‐rendering the datasets to create 3D models using Drishti (version 1.0; ANUVizlab, Canberra, Australia), followed by analysis of three different sides of the bone using ImageJ software (version 1.47; NIH, Bethesda, MD, USA; https://imagej.nih.gov/ij/). After decalcification, wax embedding, and sectioning of the tibias, the numbers of osteoclasts (following tartrate‐resistant acid phosphatase [TRAP] staining) were assessed using OsteoMeasure Advanced Bone Histomorphometry Video System (Osteometrics, Inc., Decatur, GA, USA) and standard histomorphometric methods.^28^

### Analysis of tumor burden

Tumor burden was assessed on sections of tibias that had been stained with hematoxylin and eosin, where the distinct morphology of the myeloma cells distinguishes them from normal marrow. The proportion of bone marrow occupied by the myeloma cells was assessed using OsteoMeasure and expressed as a percentage of the whole bone section area as described.^22^ Tumor burden was also assessed in bone marrow flushes of the left femora by flow cytometry using an anti‐human HLA‐ABC‐APC antibody and an isotype‐matched control (R&D Systems, Minneapolis, MN, USA) as described.^29^, ^30^

### Statistical analysis

Statistical significance was determined using the Holm‐Sidak test, a Mann‐Whitney test, or a Kruskal‐Wallis test with a Dunn's multiple comparisons test calculated using GraphPad Instat version 6.0b (GraphPad Software, Inc., La Jolla, CA, USA). All data are expressed with error bars representing standard error of the mean (SE) unless otherwise stated.

## Results

### OX14 has lower HAP binding characteristics and short‐term (24‐hour) skeletal retention dynamics than bisphosphonates currently used clinically, and is highly potent at inhibiting osteoclastic bone resorption

**Figure 1:**
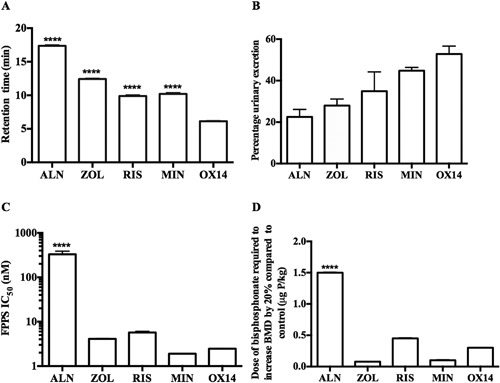
OX14 has a low bone‐binding affinity and is highly potent at inhibiting FPPS. (*A*) Retention of OX14 compared to other bisphosphonates on a HAP column over time. (*B*) Percentage urinary excretion of ALN, ZOL, RIS, MIN, and OX14 collected from 0 to 24 hours after i.v. injection. (*C*) FPPS inhibition assay of OX14 compared to other bisphosphonates. (*D*) The dose of ALN, ZOL, RIS, MIN, and OX14 (μg P/kg) that increased BMD greater than 20% compared to vehicle control in a growing rat model (proximal tibial metaphysis). Data are presented as mean ± SE (except 1B which is mean ± standard deviation) and significance compared with OX14 is indicated, where *p < 0.05, **p < 0.01, ***p < 0.001, and ****p < 0.0001.

To assess the mineral binding characteristics of OX14 compared to clinically relevant bisphosphonates, each compound was run separately on a HAP column (Fig. 1 *A*). The retention time for OX14 (6.17 ± 0.08 min) was significantly lower (*p *< 0.0001) than for ALN, ZOL, RIS or MIN.

The skeletal uptake of OX14 in vivo compared to other bisphosphonates was assessed by measuring its excretion into the urine of rats (*n* = 6) after i.v. dosing. After i.v. injection, the percentage of the dose of OX14 excreted over the ensuing 24 hours was greater than for all of the comparator bisphosphonates. The rank order for increasing excretion was ALN (22.5% ± 3.6%), ZOL (27.95% ± 3.18%), RIS (34.91% ± 9.3%), MIN (44.80% ± 1.6%), and OX14 (52.85% ± 3.82%) (Fig. 1 *B*). The numbers of animals dosed with each compound ranged from three to 42. The results obtained from these and other studies have shown that the skeletal retention of individual bisphosphonates appear to be similar, regardless of whether the drugs are given individually, in pairs, or given all together, even at different relative doses, implying that there is no significant binding competition among the bisphosphonates under the conditions used. For example, when RIS was given over a 10‐fold dose range 0.1, 0.5, and 1 mg, the % excretion was 38.1 ± 5.6, 45.1 ± 1.0, and 43.0 ± 1.8, respectively. Similar values were obtained when RIS was co‐dosed with equal or greater amounts of ALN or ZOL (43.2 ± 2.6), or with MIN or OX14 (32.5 ± 0.1).

It should be noted that these studies used doses more than 100‐fold lower than those at which skeletal saturation might be expected to happen, based on reported studies including in vitro binding studies.^31^, ^32^

Overall, the HAP binding and urinary excretion data both consistently indicated that OX14 binds less avidly to bone than the other bisphosphonates tested. These results are summarized in Fig. 1 *B* and Table 1.

**Table 1:** Summary of Data on the Bisphosphonates Studied, Showing Binding Affinities of HAP Columns, 24‐hour Skeletal Uptake in Rats, Inhibition of FPPS in Vitro, and Increases in BMD in Vivo

Bisphosphonate	Formula	HAP affinity (mean retention time/min)	Skeletal retention (% of administered dose)^a^	FPPS (IC50)^b^	Dose required to increase BMD by 20% greater than control (μg P/kg)^c^
Alendronate	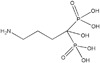	17.5 ± 0.14	77.5 ± 3.60	330.4 ± 57	1.52
Zoledronate	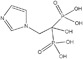	12.53 ± 0.1	72.05 ± 3.18	4.1 ± 0.07	0.08
Risedronate	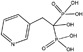	9.97 ± 0.18	65.1 ± 9.31	5.7 ± 0.4	0.45
Minodronate	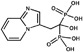	10.33 ± 0.18	55.2 ± 1.6	1.9 ± 0.01	0.11
OX14^d^	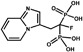	6.17 ± 0.08	47.15 ± 3.82	2.46 ± 0.01	0.30

^a^ Calculated as dose administered minus urinary excretion over 24 hours.

^b^ After preincubation.

^c^ The μg P/kg dose that increased BMD 20% greater than the control.

^d^ (1‐fluoro‐2‐(imidazo‐[1,2‐α]pyridin‐3‐yl)‐ethyl‐bisphosphonate).

The potential cellular potency of OX14 was initially assessed in an FPPS inhibition assay in vitro, where it was shown to have similar potency (IC_50_ = 2.6 nM) to ZOL, RIS, and MIN, and to be significantly more potent than ALN (*p *< 0.0001) (Fig. 1 *C*). The antiresorptive potency of OX14 was then tested in vivo and compared to ALN, ZOL, RIS, and MIN by assessing (all six mice in each group) the BMD increase in a growing rat model. OX14 was shown to have a similar potency to ZOL, RIS, and MIN, and to be significantly more potent than ALN (*p *< 0.0001) (Fig. 1 *D*). These results are summarized in Table 1.

### Evaluation of different doses of OX14 in vivo compared to clinically relevant bisphosphonates

**Figure 2:**
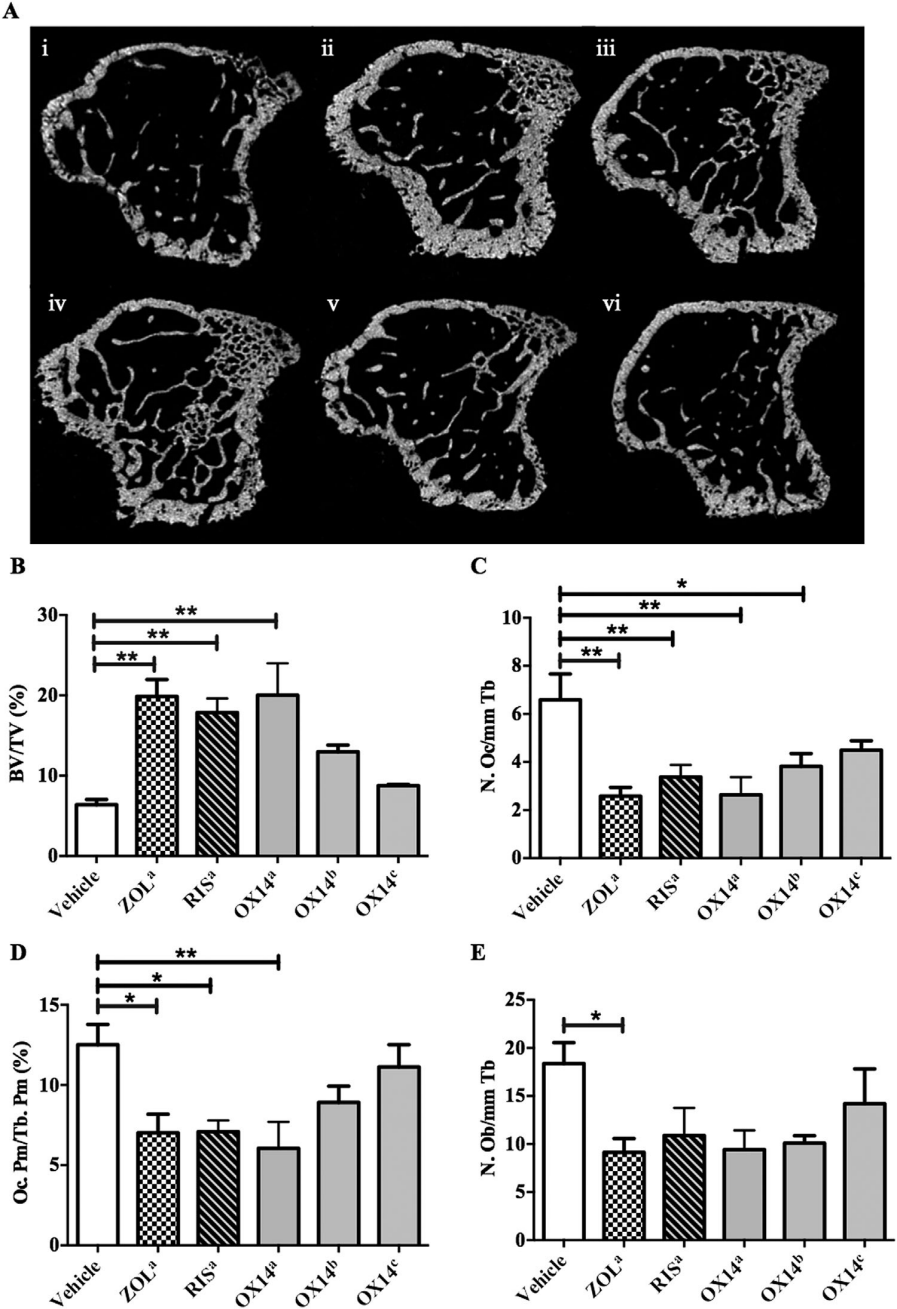
Evaluation of different doses of OX14 in vivo compared to two clinically established bisphosphonates. (*A*) Representative µCT images of transverse sections of tibias from C57Bl/6 mice treated for 3 weeks with vehicle (100 µL PBS s.c. 2×/week) (i), ZOL^a^ (28.4 µg P/kg s.c. 2×/week) (ii), RIS^a^ (27.1 µg P/kg s.c. 2×/week) (iii), or different doses of OX14^a–c^ (23, 2.3, or 0.23 µg P/kg s.c. 2×/week, and iv, v, and vi, respectively). (*B*) μCT analysis of BV/TV (%). (*C*) Histological analysis of the number of osteoclasts on the trabecular bone surfaces (Oc.N/mm Tb). (*D*) Histological analysis of osteoclast surface on the trabecular bone surfaces (Oc.Pm/Tb.Pm, %). (*E*) Histological analysis of the number of osteoblasts on the trabecular bone surfaces (Ob.N/mm Tb). Data are presented as mean ± SE and significance compared with the vehicle control, where *p < 0.05 and **p < 0.01.

In non–tumor‐bearing mice OX14 increased BV/TV as assessed (all eight mice in each group) by µCT (Fig. 2 *A*, *B*) and was as effective as ZOL and RIS when given at the same dose. However, lower doses of OX14 (2.3 and 0.23 µg P/kg) were not significantly different from the vehicle control group. Despite this, osteoclast numbers on the trabecular bone surface were significantly reduced with all compounds except for the lowest dose OX14 group (0.23 µg P/kg) when compared to the vehicle control group (Fig. 2 *C*). A similar trend was observed when the osteoclast perimeter on the trabecular bone perimeter was assessed (Fig. 2 *D*); however, neither of the lower doses of OX14 produced significant reductions compared to the vehicle control. The variability in assessment of osteoclasts, and the variability in animals, is greater than the variability in assessment of BMD. The trends are consistent with decreases in osteoclasts correlating with the increases in bone mass. In addition, we analyzed the number of osteoblasts on the trabecular bone surface (Fig. 2 *E*). A significant reduction in osteoblasts was only observed in the ZOL‐treated group. Therefore, taking all the data into account, the significant differences in BV/TV between the vehicle and bisphosphonate groups is likely to be due to the reduction of bone turnover caused by the inhibition of osteoclastic bone resorption in the bisphosphonate‐treated groups with no inhibition caused in the vehicle‐treated group.

### OX14 does not reduce tumor burden but prevents osteolytic disease in JJN3‐bearing NSG mice

**Figure 3:**
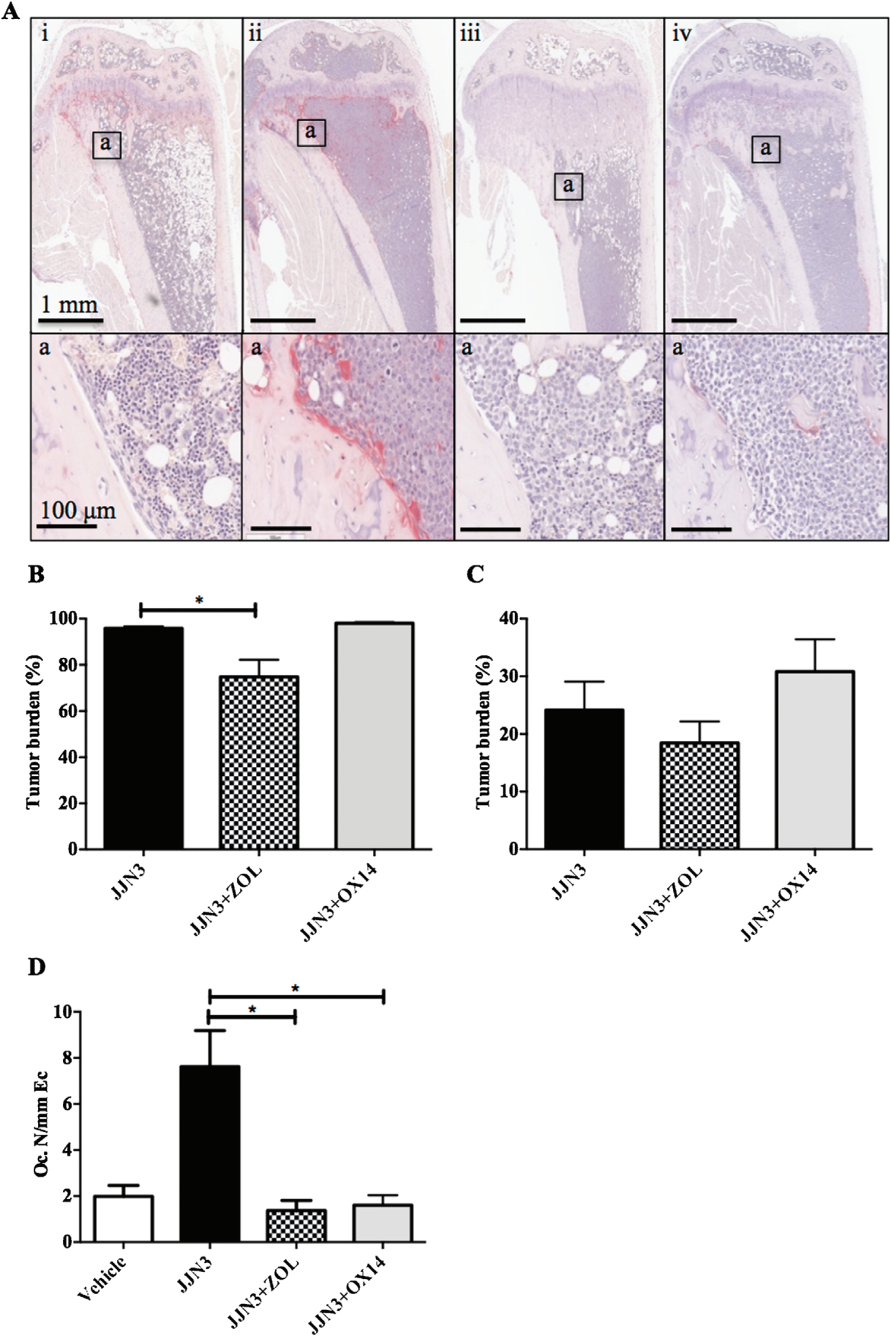
OX14 reduces osteoclast numbers in JJN3‐bearing NSG mice. (*A*) Representative histology images of longitudinal and insets of tibias from NSG mice, at the end stage of disease (3 weeks postinjection of tumor cells), injected with vehicle (naive) (i), 1 × 10^6^ JJN3 cells (JJN3) (ii) or JJN3 cells, and treated with ZOL (JJN3+ZOL) (iii) or OX14 (JJN3+OX14) (iv). Insets below show the endocortical (box a) bone regions. (*B*) Histological analysis of the percentage of tumor burden in tibias. (*C*) Flow cytometric analysis from femoral bone marrow flushes (vi) of the percentage of tumor burden. (*D*) The number of osteoclasts on the cortico‐endosteal surface (Oc.N/mm Ec). Data are presented as mean ± SE and significance from the nontumor control group (naive) is indicated, where *p < 0.05.

**Figure 4:**
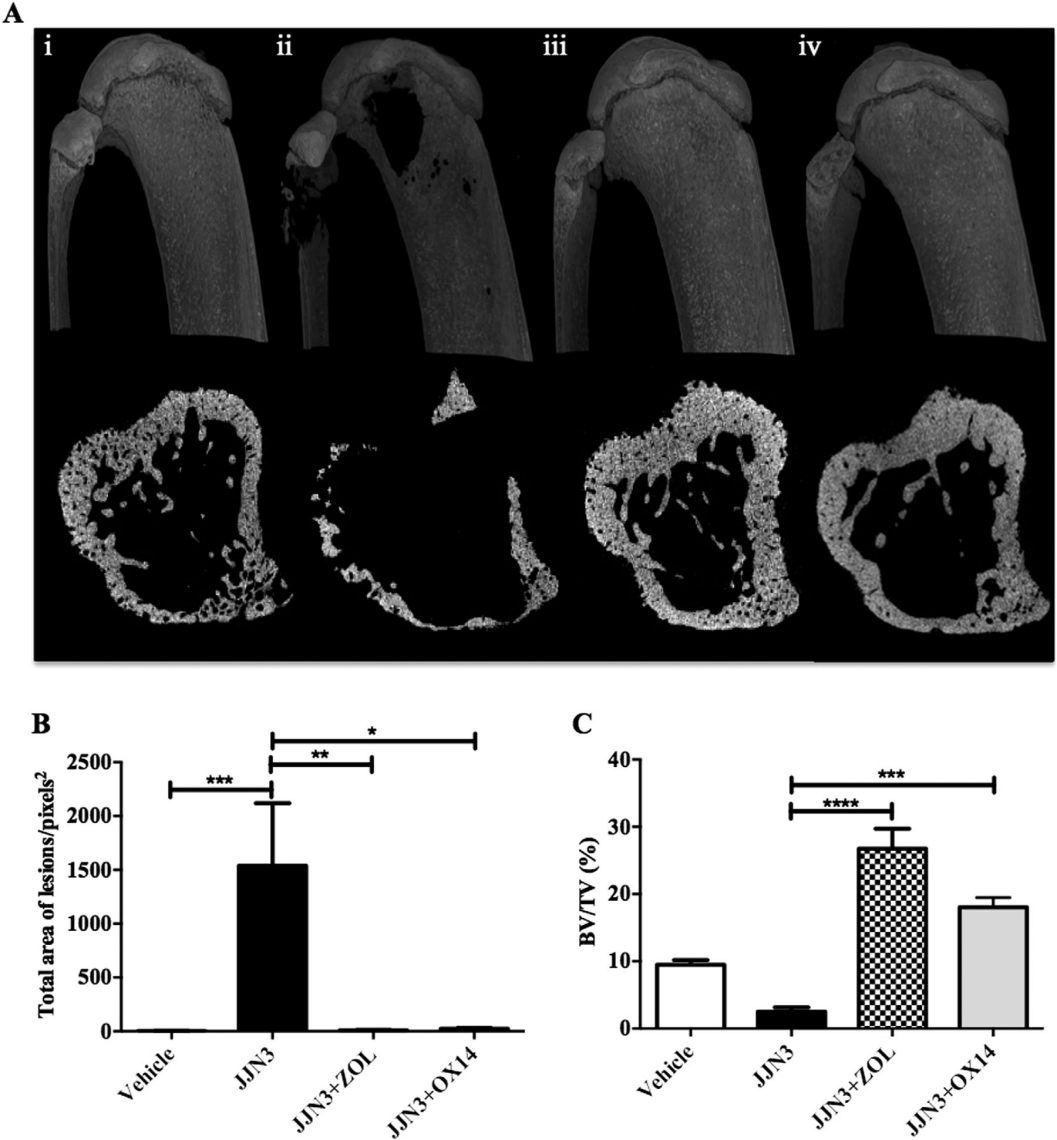
OX14 prevents osteolytic disease in JJN3‐bearing NSG mice. (*A*) Representative longitudinal and transverse µCT images of tibias from NSG mice, at the end stage of disease, injected with vehicle (naive) (i), 1 × 10^6^ JJN3 cells (JJN3) (ii) or JJN3 cells, and treated with ZOL (JJN3+ZOL) (iii) or OX14 (JJN3+OX14) (iv). (*B*) μCT analysis of the number of cortical bone lesions. (*C*) μCT analysis of BV/TV (%). Data are presented as mean ± SE and significance from the nontumor control group (naive) is indicated, where *p < 0.05, **p < 0.01, ***p < 0.001, and ****p < 0.0001.

To assess the effects of OX14 in a disease setting we treated JJN3‐bearing NSG mice with OX14 and compared its effects to ZOL (Figs. 3 and 4). We assessed (all eight mice in each group) the effect of OX14 treatment on tumor burden by histological analysis (Fig. 3 *A*i–iv, *B*) and flow cytometric analysis (Fig. 3 *C*). Histological analysis showed a significant reduction in tumor burden in the ZOL‐treated group (*p *< 0.05) compared to the tumor control group (JJN3). Interestingly, flow cytometric analysis showed no significant reduction in tumor burden with either OX14 or ZOL compared to the tumor control group (JJN3). However, a significant reduction in osteoclast number was observed when tumor mice were treated with OX14 (*p *< 0.05) or ZOL (*p *< 0.05) (Fig. 3 *D*). OX14 inhibited osteoclastic bone resorption to the same extent as ZOL, with osteoclast numbers similar to those seen in non–tumor‐bearing mice (vehicle).

μCT analysis revealed that both OX14 (*p *< 0.05) and ZOL (*p *< 0.001) prevented the osteolytic disease which was observed in the tumor control group (Fig. 4). Cortical bone lesions were significantly reduced (Fig. 4 *A*, *B*) and the BV/TV was significantly higher (Fig. 4 *C*) in tumor‐bearing mice treated with OX14 (*p *< 0.001) or ZOL (*p *< 0.0001) compared to the tumor control group.

In addition, for all the above in vivo studies no adverse side effects (loss or gain of weight, liver toxicity, or death) were observed, indicating that OX14 has a similar safety profile to other bisphosphonates.

## Discussion

In this series of studies we have evaluated the properties of OX14, a new bisphosphonate compound designed to have lower bone binding dynamics and a high antiresorptive potency.

Over the years many hundreds of different bisphosphonates have been made and more than a dozen have been registered for clinical use for various indications in various countries. One of the gratifying advances in recent years has been the elucidation of the molecular mechanisms of action through which bisphosphonates act. This has led to the recognition that the pharmacological effects of bisphosphonates as inhibitors of bone resorption appear to depend upon two key properties: their affinity for bone mineral, and their inhibitory effects on osteoclasts. Thus, although different bisphosphonates share many pharmacological properties, every bisphosphonate has a specific and often unique profile, based on its mineral binding affinity and biochemical actions. In the case of nitrogen‐containing bisphosphonates such as those studied herein, their biochemical potency is highly dependent on their inhibitory activity (IC_50_) on their main enzyme target, FPPS. The pharmacological differences among bisphosphonates may be of practical clinical importance,^33^ for example in the degree and duration of reduction of bone turnover, which may influence how long to treat patients with individual drugs.

One concept to emerge from these considerations is whether there might be value in designing compounds with somewhat reduced affinity for bone to enhance their potential direct anti‐tumor effects, while reducing their long‐term retention in bone, which has been thought to be possibly associated with adverse events. OX14 is an example of applying this concept.^34^

Here we have shown that the binding affinity of OX14 to HAP was lower than that of ALN, ZOL, RIS, and MIN. Furthermore, OX14 given to rats was excreted into the urine to a greater extent than ALN, ZOL, RIS, and MIN, indicating lower skeletal retention over the 24‐hour study period. After a single i.v. dose, there is rapid skeletal uptake within the first few hours, and this determines how much stays in the skeleton over the ensuing days and weeks.

To assess the antiresorptive potency of OX14 we used a growing rat model where it was as effective as ZOL, RIS, and MIN at increasing BMD. Similarly, in non–tumor‐bearing mice, OX14 was as effective as ZOL and RIS at increasing BV/TV. In a murine model of myeloma‐induced bone disease, OX14 was shown to prevent the development of osteolytic lesions but not to reduce tumor burden. Interestingly ZOL was shown to significantly inhibit tumor burden by histological analysis of tibial sections. However, flow cytometric analysis of tumor burden in the ZOL‐treated JJN3 mice did not show a significant reduction compared to the tumor control. Because the JJN3‐NSG model is an aggressive short‐term model, it is perhaps not that surprising that no anti‐tumor effects were observed for OX14, and perhaps a less aggressive longer‐term model would be more suitable to use to assess any nonskeletal effects of the bisphosphonates in future studies.

Interestingly, OX14 is closely related to MIN, with fluorine substituted for a hydroxyl group at the R1 position. This profoundly alters its properties in terms of reducing its mineral binding with a modest reduction in its IC_50_ on FPPS. MIN has emerged as probably the most potent bisphosphonate to be approved for clinical use by this FPPS measure, but is only used in Japan.^35^, ^36^ This illustrates how apparently small differences in chemical structure can produce large changes in pharmacological properties, as has been observed with many other apparently closely related bisphosphonates.

There is an increasing realization that bisphosphonates have an extensive range of biological actions in addition to their well‐established effects on bone resorption. Examples of observed clinical benefits outside the field of bone diseases include a reduction in mortality observed with ZOL in the Horizon trial.^16^, ^17^ Other observational studies have shown a reduction in mortality after hip fracture in patients receiving oral bisphosphonates,^15^ a reduction of myocardial infarctions in rheumatoid arthritis patients treated with bisphosphonates,^18^ and improved survival of patients who had received a bisphosphonate prior to admission to an intensive care unit.^19^ Administration of oral bisphosphonates may also be associated with a reduction in deaths from colon cancer.^20^, ^21^ The pharmacology underlying these potential effects of bisphosphonates needs to be understood, but in theory many of these effects are likely to result from the known inhibitory effects of bisphosphonates on the mevalonate pathway and the consequent effects on protein prenylation and intracellular signaling pathways in key tissues. For example, the ability of bisphosphonates to prolong the survival of mesenchymal stem cells and to enhance DNA repair after irradiation are dependent on the mevalonate pathway.^37^

Bisphosphonates have an overall excellent safety profile. The development of new and even more potent compounds with lower bone‐binding dynamics, such as OX14, offer promising opportunities for use in these emerging and novel applications. A key question will be whether the lower bone‐binding properties will offer real pharmacological advantages.

One recent example of the potential utility of nonskeletal effects of OX14 is its effectiveness in preventing experimentally induced inflammatory colitis.^38^

In summary, OX14 is a new, highly potent bisphosphonate with reduced bone binding properties compared with other clinically relevant bisphosphonates. This renders OX14 an attractive candidate for clinical development for its potential skeletal or nonskeletal benefits. A full evaluation of its potential will require further studies of its possible uses and safety profile.

## Disclosures

MWL and FHE are consultants for Biovinc. RGGR has acted as legal expert for Novartis, Merck, and Lilly.

## Supporting information

Supporting Information.
