# Supplementary material for: The Pharmacological Profile of a Novel Highly Potent Bisphosphonate, OX14 (1‐Fluoro‐2‐(Imidazo‐[1,2‐α]Pyridin‐3‐yl)‐Ethyl‐Bisphosphonate)
Source: J Bone Miner Res. 2017 Apr 21;32(9):1860–9. doi: 10.1002/jbmr.3138 (PMC5596338; doi:10.1002/jbmr.3138)
Supplement: Supplementary file 1 — Supporting Information. [file JBMR-32-1860-s001.docx]

**Supporting Materials and Methods**

**Synthesis of OX14: 1-fluoro-2-(imidazo-[1,2-α]pyridin-3-yl)ethyl-bisphosphonate**

*3-iodo-imidazo-[1,2*-α*]pyridine*

A solution of 11.61 g (98.27 mmol) of imidazopyridine in 100 ml of acetonitrile was placed in a 250 ml three-neck round-bottom flask equipped with a magnetic stirrer, thermocouple, nitrogen bleed, and cooling ice bath. A total of 24.32g (108.1 mmol, 1.1 *eq*.) of solid N-iodosuccinimide was added portion-wise to the flask at 0^o^C, and the resulting yellow suspension was allowed to warm to room temperature overnight. The solvent was removed on rotavap to give the dark solid (39.2 g). This residue was re-dissolved in 0.5 L of dichloromethane and washed with 10% KOH (2 x 250 ml). The organic phase was separated, washed with water (200 ml), and dried over Na_2_SO_4_. The solvent was removed on rotavap, and the remaining dark material was re-crystallized from boiling ethyl acetate upon the addition of hexane. The precipitated solid was filtered, washed with 40 ml of hexane, and dried under nitrogen flow until constant weight. The yield of *3-iodo-imidazo-[1,2-*α*]pyridine* was 20.8 g (86%) of white solid with R_f_ = 0.42 (in EtOAc/hexane = 1:1).

H^1^ NMR (CDCl_3_), δ: 8.12 (d, 1H, J=6.8 Hz), 7.69 (s, 1H), 7.62 (d, 1H, J=6.8 Hz), 7.24 (t, 1H, J=6.8 Hz), 6.94 (t, 1H, J=6.8 Hz).

^13^C NMR (CDCl_3_), δ: 147.52, 140.02, 126.08, 125.23, 117.76, 113.43, 60.91. LC-MS (ESI) for C_7_H_5_N_2_I m/z 245 [M+H]^+^.

*Tetraethyl-2-(imidazo-[1,2*-α*]pyridin-3-yl)-ethyl-bisphosphonate*

A solution of 6.67 g (27.31 mmol) of 3-iodo-imidazo-[1,2-α ]pyridine in 200 ml of THF was placed in a 0.5 L 3-neck round bottom flask equipped with a magnetic stirrer, thermocouple, nitrogen bleed, and cooling dry ice/acetone bath. The solution was chilled on a dry ice/acetone bath, and n-BuLi (2.5M/hexane, 22 ml, 2 *eq*.) was gradually added at -74^o^C to the white suspension (that resulted due to poor solubility of 3-iodo-imidazo-[1,2-α]pyridine at low temperature). During the addition of n-BuLi some sticky white material precipitated on the flask’s walls, thus resulting in obstruction of stirring. After twenty minutes a total of 8.15 g (27.14 mmol) of the vinyl phosphonate, tetraethyl ethene-1,1-bisphosphonate (prepared according to literature procedures *J. Org. Chem.*, 51: 3488-3490 (1986)), in 20 ml of THF was introduced into a flask at -75^o^C. A sticky semisolid gradually dissolved, and the color of the reaction mixture turned almost black. After 30 minutes of stirring at -75^o^C, the reaction was allowed to gradually warm to -40^o^C and then was quenched by slow addition of 200 ml of saturated NH_4_Cl. The resulting mixture was warmed to ambient temperature, the organic layer was separated, and the aqueous phase extracted with ethyl acetate (3 x 100 ml). The combined organic phases were washed with brine (100 ml), dried over Na_2_SO_4,_ and concentrated on rotavap to give 8.1 g of dark oil. This oil was purified by CombiFlash on silica gel and after concentration of pure fractions (purity was checked by ^31^P NMR) a total of 1.7 g (15% yield) of pure tetraethyl-2-(imidazo-[1,2-α ]pyridin-3-yl)-ethyl-bisphosphonate was isolated as a dark oil with R_f_ = 0.18 in EtOAc/MeOH = 8:2.

^1^H NMR (CDCl_3_), δ: 8.23 (d,1H, J=7.2 Hz), 7.60 (d, 1H, J=9.2 Hz), 7.53 (s,1H), 7.16 (dd, 1H, J=9.2, J=7.2 Hz), 6.84 (t, 1H, J=6.8Hz), 4.11 (m, 8H), 3.57 (td, 2H, ^3^J_HH_=6.2, ^3^J_PH_=15.6 Hz), 2.63 (tt, 1H, ^3^J_HH_=6.2, ^2^J_PH_=23.6 Hz), 1.27 (q, 12H, ^3^J_HH_=7.2 Hz).

^13^C NMR (CDCl_3_), δ: 145.3, 133.0, 123.55, 123.52, 121.3 (t, ^3^J_CP_=8.0 Hz), 117.9, 112.1, 62.9 (d, ^2^J_COP_=6.6 Hz), 62.7 (d, ^2^J_COP_=6.6 Hz), 36.1 (t, J_CP_=132.8 Hz), 20.0 (t, ^2^J_CP_=4.8 Hz), 16.3, 16.2.

^31^P NMR (CDCl_3_), δ: 22.2. LC-MS (ESI) for C_17_H_28_N_2_O_6_P_2_ m/z 419 [M+H]^+^.

*Tetraethyl-1-fluoro-2-(imidazo-[*1,2-α*]pyridin-3-yl)-ethyl-bisphosphonate*

A 30% suspension of KH in paraffin oil (0.9965 g, 7.45 mmol, 1.83 *eq*.) was charged in a 50 ml three-neck round bottom flask equipped with a magnetic stirrer, thermocouple, nitrogen line, and cooling bath. Anhydrous THF (15 ml) was added into the flask, and the resulting suspension was chilled to 0^o^C. A solution of the bisphosphonate tetraethyl-2-(imidazo-[1,2-α]pyridin-3-yl)-ethyl-bisphosphonate (1.6955 g, 4.05 mmol) in 15 ml of THF was slowly added to the suspension of KH, and the resulting brown-colored solution was brought to ambient temperature in 30 minutes. A catalytic amount of 18-crown-6 (0.3275 g, 1.23 mmol, 30 mol%) was added to the reaction, and after 5 minutes of stirring the mixture was chilled to 0^o^C. A total of 3.0785 g (8.68 mmol, 2.1 *eq.*) of Selectfluor*^®^* was added portion wise at 0^o^C, and the mixture stirred at this temperature until all starting material was consumed (about 3-5 hours). The reaction was monitored by TLC (EtOAc/MeOH = 8:2), and after completion it was quenched with ice-cold saturated NaHCO_3_ solution (50 ml). The product was extracted with ethyl acetate (3 x 50 ml), and the combined organic phases were washed with brine (30 ml) and dried over Na_2_SO_4_. After concentration under reduced pressure a total of 1.92 g of dark oil was obtained. This crude material was purified on silica gel using ethyl acetate and ethyl acetate/MeOH = 9:1 mixtures as eluent to give 1.164 g of clear amber oil. According to ^31^P NMR, this oil was contaminated with an elimination product possessing essentially the same R_f_ value as the desirable product tetraethyl-1-fluoro-2-(imidazo-[1,2-α]pyridin-3-yl)-ethyl-bisphosphonate. Therefore, it was further purified by preparative HPLC (a total of 6 runs were completed). After concentration of “good” fractions and freeze-drying a total of 0.6791 g (38% yield) of pure tetraethyl-1-fluoro-2-(imidazo-[1,2-α]pyridin-3-yl)-ethyl-bisphosphonate was isolated.

**OX14: *1-fluoro-2-(imidazo-[1,2*-α*]pyridin-3-yl)ethyl-bisphosphonate***

A solution of tetraethyl-1-fluoro-2-(imidazo-[1,2-α]pyridin-3-yl)-ethyl-bisphosphonate (0.6465 g, 1.48 mmol) in 10 ml of anhydrous chloroform was placed in a 50 ml 3-neck round bottom flask equipped with a magnetic stirrer, thermocouple, condenser, heating mantle, and nitrogen bleed. Neat TMSBr (2.0973 g, 13.69 mmol, approximately 9 eq.) was added to this solution at room temperature, and the resulting solution stirred at 50° C. until only the mass M−1=323 was observed in LC-MS in negative mode (usually complete de-protection requires 20-24 hours). The reaction mixture was cooled to room temperature and concentrated on rotavap to give 0.6887 g of yellow oil. This oil was dissolved in 5 ml of anhydrous chloroform and filtered through the 0.45 μm PTFE membrane filter to give a clear yellow solution. A total of 5 ml of DI water was slowly added to this solution followed by slow addition of acetonitrile (about 15 ml) until crystallization occurred. The resulting suspension was stirred for 30 minutes at room temperature and then filtered through a fine sintered glass filter. The isolated solid was washed with water (2×3 ml) followed by methanol (2×3 ml) and anhydrous ether (2×5 ml) washes. After drying under suction and nitrogen blanket the white solid was additionally dried overnight in a drying apparatus over phosphorous pentoxide under vacuum (1.5 mm Hg) at 40° C. The yield was 240 mg (50%) of OX14 (**1-fluoro-2-(imidazo-[1,2-α]pyridin-3-yl)ethyl-bisphosphonate)** as a white solid, having the following structure:

Elemental analysis (%) calculated for C_9_H_11_FN_2_O_6_P_2_.0.5H_2_O (333.15): C 32.45, H 3.63, N 8.41. Found C 32.39, H 3.54, N 8.23. ^1^H NMR (300 MHz, D_2_O+KOD, ppm): 3.76 (tt, 2H, J=21.6, 11.7), 6.92 (t, 1H, J=6.6 Hz), 7.28 (m, 1H), 7.59-7.47 (m, 2H). ^13^C NMR (75 MHz, D_2_O+KOD, ppm): 28.64 (d, J=18.8), 87.63, 99.43, 100.0, 101.23, 101,81, 103.60, 111.88, 115.22, 123.15, 123.23, 124.98, 126.25, 126.31, 132.07, 144.92. ^31^P NMR (121 MHz, D_2_O+KOD, ppm): 13.57 (d, J=66 Hz).^19^F NMR (282 MHz, D_2_O+KOD, ppm): −182.36 (m, J=67 Hz, 21 Hz). MS(ESI) 323 (M−1).
